# Transgenerational effects of inter-ploidy cross direction on reproduction and F2 seed development of *Arabidopsis thaliana* F1 hybrid triploids

**DOI:** 10.1007/s00497-019-00369-6

**Published:** 2019-03-21

**Authors:** Dorota Duszynska, Bjarni Vilhjalmsson, Rosa Castillo Bravo, Sandesh Swamidatta, Thomas E. Juenger, Mark T. A. Donoghue, Aurélie Comte, Magnus Nordborg, Timothy F. Sharbel, Galina Brychkova, Peter C. McKeown, Charles Spillane

**Affiliations:** 1 Genetics and Biotechnology Laboratory, Plant and AgriBiosciences Research Centre (PABC), Ryan Institute, National University of Ireland, Galway, H91 REW4 Ireland; 2 Gregor Mendel Institute of Molecular Plant Biology, Vienna, Austria; 3 Section of Integrative Biology and Institute for Cellular and Molecular Biology, University of Texas, Austin, USA; 4 Apomixis Research Group, Department of Cytogenetics and Genome Analysis, Leibniz Institute of Plant Genetics and Crop Plant Research (IPK), Gatersleben, Germany; 5 Present Address: Bioinformatics Research Centre, Aarhus University, Århus, Denmark; 6 Present Address: Department of Biology, Centre for Novel Agricultural Products (CNAP), University of York, York, UK; 7 Present Address: Memorial Sloan Kettering Cancer Center, New York, NY USA; 8 Seed and Developmental Biology Program, Global Institute for Food Security, University of Saskatchewan, Saskatoon, SK S7N 4J8 Canada

**Keywords:** F1 hybrid, Triploid bridge, Transgenerational effect, Epigenetic, Reproduction, Plant evolution

## Abstract

**Key message:**

Reproduction in triploid plants is important for understanding polyploid population dynamics. We show that genetically identical reciprocal F1 hybrid triploids can display transgenerational epigenetic effects on viable F2 seed development.

**Abstract:**

The success or failure of reproductive outcomes from intra-species crosses between plants of different ploidy levels is an important factor in flowering plant evolution and crop breeding. However, the effects of inter-ploidy cross directions on F1 hybrid offspring fitness are poorly understood. In *Arabidopsis thaliana*, hybridization between diploid and tetraploid plants can produce viable F1 triploid plants. When selfed, such F1 triploid plants act as aneuploid gamete production “machines” where the vast majority of gametes generated are aneuploid which, following sexual reproduction, can generate aneuploid swarms of F2 progeny (Henry et al. 2009). There is potential for some aneuploids to cause gametophyte abortion and/or F2 seed abortion (Henry et al. 2009). In this study, we analyse the reproductive success of 178 self-fertilized inter-accession F1 hybrid triploids and demonstrate that the proportions of aborted or normally developed F2 seeds from the selfed F1 triploids depend upon a combination of natural variation and cross direction, with strong interaction between these factors. Single-seed ploidy analysis indicates that the embryonic DNA content of phenotypically normal F2 seeds is highly variable and that these DNA content distributions are also affected by genotype and cross direction. Notably, genetically identical reciprocal F1 hybrid triploids display grandparent-of-origin effects on F2 seed set, and hence on the ability to tolerate aneuploidy in F2 seed. There are differences between reciprocal F1 hybrid triploids regarding the proportions of normal and aborted F2 seeds generated, and also for the DNA content averages and distributions of the F2 seeds. To identify genetic variation for tolerance of aneuploidy in F2 seeds, we carried out a GWAS which identified two SNPs, termed *MOT* and *POT*, which represent candidate loci for genetic control of the proportion of normal F2 seeds obtained from selfed F1 triploids. Parental and grandparental effects on F2 seeds obtained from selfed F1 triploids can have transgenerational consequences for asymmetric gene flow, emergence of novel genotypes in polyploid populations, and for control of F2 seed set in triploid crops.

**Electronic supplementary material:**

The online version of this article (10.1007/s00497-019-00369-6) contains supplementary material, which is available to authorized users.

## Introduction

Triploidy is predicted to occur commonly during flowering plant evolution due to the fusion of spontaneously occurring unreduced diploid gametes with wild-type haploid gametes, from polyspermy (Nakel et al. 2017), or from inter-ploidy hybridization between diploid and tetraploid individuals within a population (Ramsey and Schemske 1998). Many flowering plants tolerate polyploidy well (Leitch and Bennett 1997; Comai 2005), and polyploidy can contribute to heterosis effects (Fort et al. 2016). In some plants, the F1 seeds generated from inter-ploidy crosses abort due to endosperm imbalance in the F1 seed (Bomblies and Weigel 2007; Köhler et al. 2010; Scott et al. 1998a; Grossniklaus et al. 2001). However, in species such as the model eudicot *Arabidopsis thaliana* (L.) Heynh., Brassicaceae (hereafter “Arabidopsis”), crosses between diploids and tetraploids can generate viable F1 triploid offspring (Henry et al. 2005; Scott et al. 1998b). Such F1 triploids can allow gene flow between naturally occurring populations with different ploidy levels if they backcross with their diploid or tetraploid progenitors, thus acting as evolutionary bridges between ploidy levels (Husband 2004).

The evolutionary importance of such triploid bridges is complicated by the fact that F1 triploids produce swarms of aneuploid F2 offspring when self-fertilized (Henry et al. 2009). Meiosis in F1 triploids generates a swarm of aneuploid gametes which in turn (following fertilization) generate a swarm of aneuploid F2 seeds. Depending on the extent of tolerance of the species to different aneuploid genotypes, gametophyte and/or F2 seed abortion can occur. Hence, a key determinant of whether populations can exchange genes via triploid intermediaries is the ability of their seeds to tolerate aneuploidy, as classically demonstrated by McClintock’s seminal work on triploid cereals (McClintock 1929). Because of the existence of naturally occurring polyploid populations and its tolerance to triploidy, Arabidopsis is a useful model for investigating the genetic control of aneuploidy tolerance in F2 progeny generated from F1 triploid plants.

To identify genes associated with such aneuploidy tolerance, Henry and colleagues used a population of recombinant inbred lines (RILs) made from crosses between the tetraploid Arabidopsis accession Wa-1 and diploid Col-0 (Schiff et al. 2001) and identified a locus on the arm of chromosome 1 as a determinant of triploid offspring production (Henry et al. 2007; Aherne et al. 2010). This QTL, which has not been mapped to any gene, was shown to be associated with tolerance to aneuploidy in the offspring of self-fertilized Arabidopsis triploids (although not in backcrosses to diploid or tetraploid parents) and was termed *sensitive to dosage imbalance* (*SDI*) accordingly (Henry et al. 2009). The activity of *SDI* was interpreted in terms of the aneuploidy selection index (ASI) acting upon the swarm of progeny from self-fertilized F1 triploids.

There is also some evidence that aneuploidy tolerance can differ between varieties of the same species, as occurs between cultivars of tomato and barley (Rick and Notani 1961; Ramage 1960). This suggests that ASI might be subject to natural variation and that different Arabidopsis F1 hybrid triploid genotypes may vary in their reproductive success when selfed. We have previously demonstrated that natural variation affects the reproductive success of selfed F1 hybrid triploids with respect to both the fertility and number of ovules (Duszynska et al. 2013). Our previous study discovered an unexpected parental effect, where the direction of the parental inter-ploidy cross used to generate the F1 triploid displayed a parental effect on the ovule fertility of the F1 hybrid when self-fertilized. We argued that this parental effect could be due to long-lasting maternal factors, uniparental inheritance of cytoplasmic organelle genomes, epigenetic differences between maternally and paternally inherited chromosomes, or some combination of these.

The influence of natural genetic variation on post-fertilization reproductive success (i.e. F2 seed production) from selfed F1 hybrid triploids has not previously been determined.

**Fig. 1:**
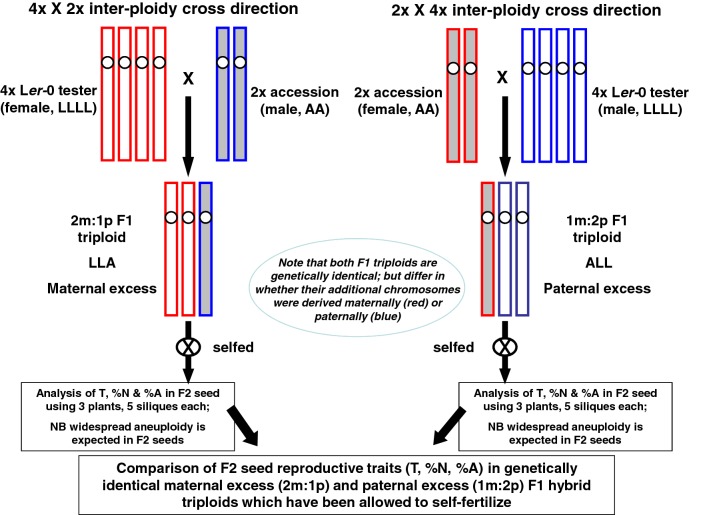
Crossing scheme used to generate F1 hybrid triploids. Illustration of the crossing scheme used to generate genetically identical pairs of reciprocal F1 hybrid triploids, which were selfed to generate F2 seed set. Each diploid (2×) accession (AA) was reciprocally crossed with a Ler-0 tetraploid (LLLL) to generate two genetically identical F1 hybrid triploids, with either two sets of maternally inherited chromosomes (2m:1p) or two sets of paternally inherited chromosomes (1m:2p). Note that the reciprocal F1 hybrids are genetically identical, differing only in the parent-of-origin of the chromosome sets (i.e. LLA vs ALL). Chromosomes of the L*er*-0 tester line (genotype LLLL) are represented by white-shaded boxes, while those of the diploid accession (example genotype AA) are grey-shaded. Maternally derived chromosome sets are outlined in red, while paternally derived chromosome sets are highlighted in blue. Each reciprocal F1 hybrid triploid plant was selfed, and the total number of ovules was measured (i.e. T) along with the proportion of F2 seeds, along with the number of normal versus aborted F2 seeds

To determine the impact of natural variation on F2 seed production from selfed F1 hybrid triploid plants, we conducted reciprocal crosses between a tetraploid tester line and 88 genetically different Arabidopsis accessions to generate 178 F1 hybrid triploid genotypes which were selfed to generate F2 seed offspring. As the F1 hybrid triploids generated from each reciprocal cross are genetically identical, our reciprocal cross design allowed us to test whether the original cross direction used to generate an F1 hybrid triploid had an effect on the F2 seed production from the F1 hybrid triploid when subsequently selfed (Fig. 1). We identified the factors controlling post-fertilization reproductive success of selfed triploid plants by characterizing the effects of genotype and parental cross direction on F2 seed development from self-fertilized Arabidopsis F1 hybrid triploids. Although many F2 seeds abort, we demonstrate that successful F2 seed development is possible from across the 178 F1 hybrid triploid genotypes tested and that the extent of such F2 seed development is affected by natural variation. Strikingly, the direction of the original inter-ploidy cross that generated the F1 triploid hybrid also affects the post-fertilization F2 seed development. This indicates that parental genome imbalance effects generated via reciprocal inter-ploidy crosses that produce genetically identical F1 hybrid triploids can in turn have transgenerational “grandparental” -type effects on the development of F2 seed generated from the selfed F1 hybrid triploids.

## Results

### Genotypic effects on F2 seed abortion in Arabidopsis F1 hybrid triploids

To determine whether parental genome dosage effects on seed production were manifest in F2 seed production from a range of different genotypes of selfed F1 triploids, we generated a series of F1 hybrid triploid *A. thaliana* plants by crossing a tetraploid tester line of accession L*er*-0 with diploid plants of each of 88 genetically different accessions. Reciprocal crosses were performed in both directions as the phenotypes of F1 seed produced from reciprocal inter-ploidy crosses are known to vary by cross direction (Berger and Chaudhury 2009; Scott et al. 1998a). The F1 hybrid triploid plants produced by each reciprocal cross combination are genetically identical, each possessing two sets of chromosomes from L*er*-0 and the other set from a genetically different diploid parent (Fig. 1). Each reciprocal F1 hybrid triploid pair therefore differs in relation to whether the two sets of L*er*-0 chromosomes are inherited maternally or paternally, and similarly for the set of chromosomes from the diploid parent (Fig. 1). We have previously demonstrated that these F1 triploid lines are euploid (Duszynska et al. 2013), but as explained in Introduction we expect their F2 progeny to be aneuploid due to the generation of aneuploid gametes arising from triploid meiosis. Hence, the ability of selfed F1 Arabidopsis triploids to produce viable F2 seeds will depend upon their ability to enhance the numbers of euploid gametes resulting from triploid meiosis, and/or to tolerate aneuploidy during subsequent gametogenesis, fecundation and post-fertilization seed development.

**Fig. 2:**
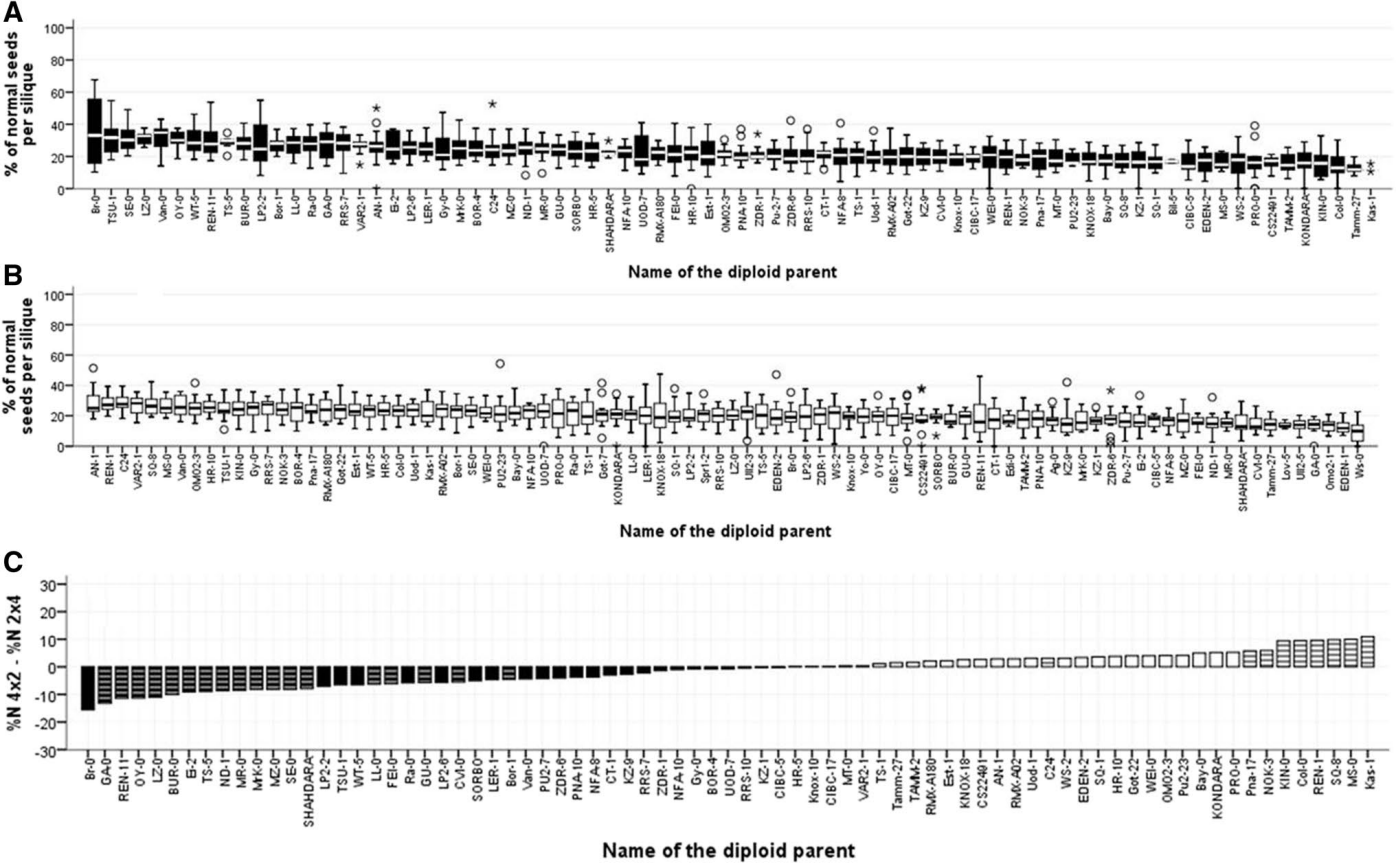
Variation in phenotypically normal F2 seed development in F1 hybrid Arabidopsis triploids. 88 pairs of genetically identical F1 hybrid triploids were generated by reciprocally crossing diploid accessions with a L*er*-0 tetraploid tester line and allowed to self-pollinate to produce F2 seeds. Proportion of phenotypically normal F2 seeds (*N*) was calculated as a proportion of the total number of ovules (*T*) per silique such that %*N* = *N*/*T* and displayed as box-and-whisker plots (showing median, inter-quartile range and outliers (circles, stars)), arranged along the x-axis from greatest to least %*N*. Box plots for %*N* produced by paternal excess hybrids (1m:2p) are shaded (**a**), those from maternal excess hybrids (2m:1p) are white (**b**). The differences between the mean values were also calculated %*N*2m:1p−%*N*2m:1p and arranged in ascending order (**c**) with reciprocal pairs of triploids that produced greater %*N* from 1m:2p F1 triploids shaded and those producing greater %*N* from 2m:1p F1 triploids white; bars marked with horizontal lines are those with significant differences between the reciprocal triploid pairs (*t* test, *p* < 0.05 after adjustment for multiple testing)

**Fig. 3:**
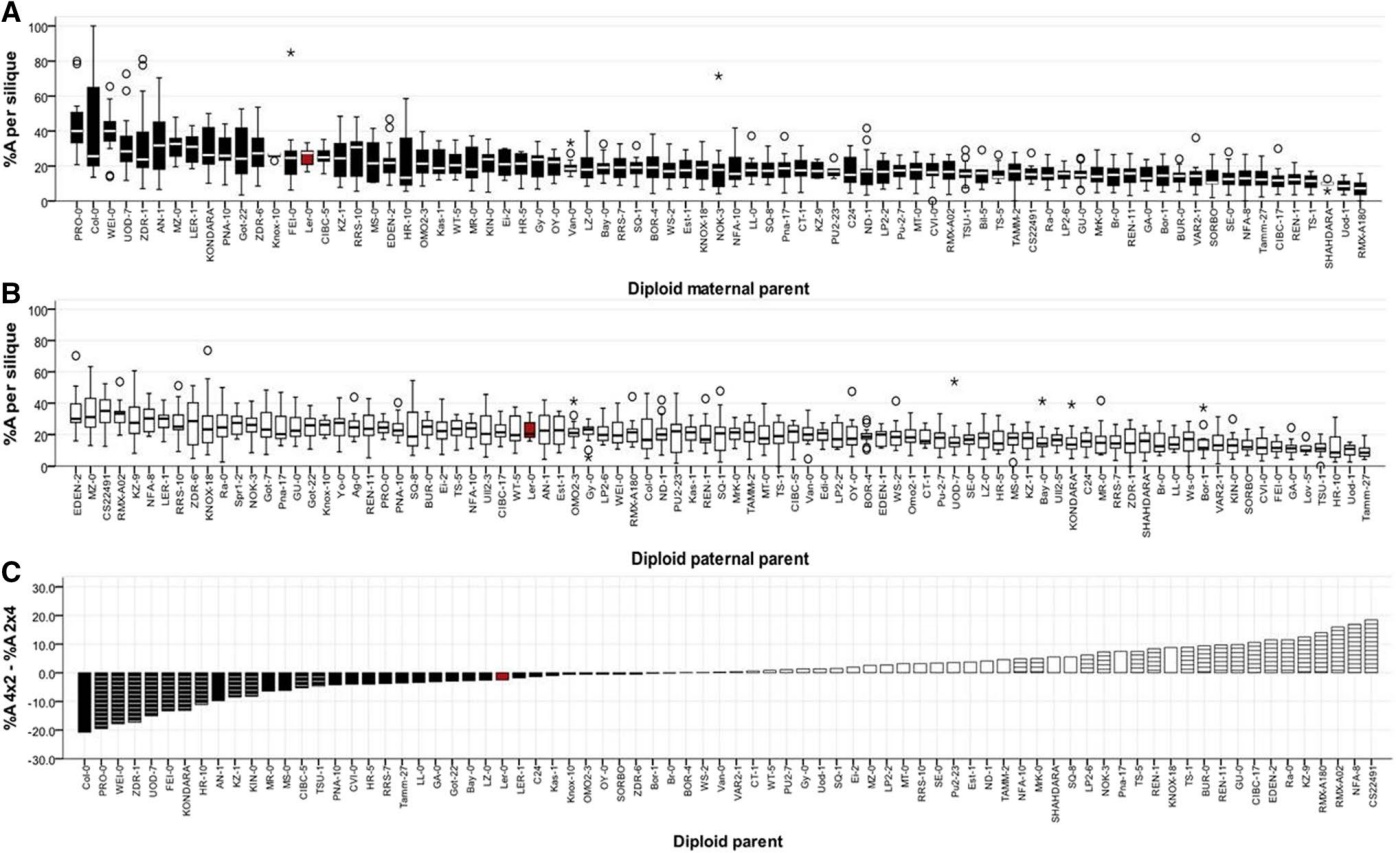
Variation in extent of post-zygotic F2 seed abortion in F1 hybrid Arabidopsis triploids. 88 pairs of genetically identical F1 hybrid triploids were generated by reciprocally crossing diploid accessions with a L*er*-0 tetraploid tester line and allowed to self-pollinate to produce F2 seeds. Proportion of aborted F2 seeds (*A*) was calculated as a proportion of the total number of ovules (*T*) per silique such that %*A* = *A*/*T* and displayed as box-and-whisker plots (showing median, inter-quartile range and outliers (circles, stars)), arranged along the x-axis from greatest to least %*N*. Box plots for %*A* produced by paternal excess hybrids (1m:2p) are shaded (**a**), those from maternal excess hybrids (2m:1p) are white (**b**). The differences between the mean values were also calculated %*N*2m:1p−%*N*2m:1p and arranged in ascending order (**c**) with reciprocal pairs of triploids that produced greater %*N* from 1 m:2p F1 triploids shaded and those producing greater %*N* from 2m:1p F1 triploids white; bars marked with horizontal lines are those with significant differences between the reciprocal triploid pairs (*t* test, *p* < 0.05 after adjustment for multiple testing)

To determine the effect of natural variation, each of the F1 hybrid triploid plants was selfed to produce F2 seeds, which were scored for the proportions of normally developing (%*N*) versus aborted (%*A*) F2 seeds as described in Materials and Methods [following (Meinke 1994)], with aborted seeds scored as those with a shrunken appearance, brown colouration and reduced size (Figure S1). We have previously presented the extent of variation in the percentages of unfertilized ovules from the selfed F1 hybrid triploid plants (Duszynska et al. 2013), determining significant variation in ovule number and fertilization rate in both paternal and maternal excess triploids, and a significant trade-off between ovule number and fertility. In this study, we detected significant variation in both %*N* and %*A* F2 seeds generated from selfed F1 hybrid triploids (Figs. 2 and 3, Table S1A and S1B). We highlight that the total F2 seed production values of %*A* and %*N* are not entirely dependent on each other, as they were calculated as a proportion of the total numbers of ovules, some of which were not successfully fertilized (%*U*). Hence, different F1 hybrid triploid genotypes displayed variation in both abortion rate and normal F2 seed production. To account for this variation due to the proportion of the ovules which remained unfertilized, we also determined the proportions of normally developed and aborted seeds as a total of those fertilized %*N*/(*A* + *N*) and %*A*/(*A* + *N*), respectively (Figure S2; Table S1C).

Post-fertilization F2 seed abortion [whether expressed as %*A* or %*A*/(*A* + *N*)] was much higher in F1 hybrid triploids than in F1 hybrid diploid Arabidopsis, in which nearly all fertilized seeds develop without abortion (Figure S3), while the %*N* of F2 seeds was reduced in the F1 hybrid triploids (Figs. 2, 3 and S2). However, the extent of post-fertilization F2 seed abortion varied between the different F1 hybrid triploid genotypes: for example, over 76% of fertilized ovules formed normal F2 seed in paternal excess F1 hybrid triploids generated from Rmx-A180, compared to under 28% of normal F2 seeds for the paternal excess F1 hybrid triploids generated from Pro-0 (Table S1D).

### “Grandparental-type” parent-of-origin effect on F2 seed development from genetically identical reciprocal F1 hybrid triploids

The reciprocal inter-ploidy crosses between the L*er*-0 tetraploid tester line and each of the diploid accessions generated pairs of F1 hybrid triploids that are genetically identical, but differ according to whether sets of chromosomes from each parent are maternally or paternally inherited (Fig. 1). We found that the proportions of normally developing and aborted F2 seeds varied between the genetically identical pairs of reciprocal F1 hybrids, as well as between F1 hybrid triploid genotypes (Table S1C). This indicates that the reproductive success of F2 seeds is not purely due to the parental F1 triploid genotype, but is also affected by the direction of the cross through which the F1 triploid parental plant was itself formed, as we have previously observed with the fertilization rate and ovule number (Duszynska et al. 2013). The value of this grandparental effect again varied between genotypes, with an average divergence of 9.79% for %*N*/(*A* + *N*), equating to 18.1% or 19.2% of the average value of this trait in F2 seed offspring of 1m:2p and 2m:1p F1 triploids, respectively (Table S1C).

To investigate this grandparental-type effect on F2 seed production and its interaction with genotype more fully, we determined the sources of heritable variation. Highly significant heritable variation was detected for both %*N* and %*A* F2 seed production (Table 1; Figure S4), with broad-sense heritabilities ranging from 0.27 to 0.44 (*p* < 0.0001). This indicates that the impact of variation between F1 hybrid triploid individuals (through, for example, any undetected local aneuploidies) or from environmental interactions effects was relatively minor, as expected given the randomized plant growth pattern followed. Although support for an effect of parental cross direction per se was much more limited (*p* < 0.1), a major effect for the accession × cross direction interaction effect was detected (*p* < 0.001). Accordingly, the response to cross direction clearly varied between genotype, both in terms of whether there was a parent-of-origin effect and in terms of its direction. In total, parent-of-origin effects were statistically significant for 29 pairs of reciprocal F1 hybrid triploids in relation to post-fertilization F2 seed abortion (%*A*) and for 27 pairs in relation to normal F2 seed development (%*N*) (Figs. 2c, 3c). We found that there was no significant correlation between total F2 ovule number (T) and %*N* in either cross directions (*p* > 0.05), but a significant negative correlation was found to exist between the proportion of ovules fertilized and the proportion which subsequently developed F2 seeds without aborting (1m:2p, *p* = 0.001; 2m:1p, *p* = 0.002). If causal, this relationship could suggest that when more ovules are successfully fertilized the risk of subsequent F2 seed abortion is greater, suggesting that resource commitment is a barrier to successful F2 seed production from F1 triploid reproduction. Full values for the phenotypic and genetic correlations between traits are presented in Table 2.

**Table 1:** Broad-sense heritabilities of the total number of ovules per silique (*T*) produced by F1 triploids with maternal or paternal genome excess, and the proportions of aborted and normal F2 seeds (%*A* = *A*/*T*, %*N* = *N*/*T*)

Trait	Genotype	
	Maternal excess (2m:1p)	Paternal excess (1m:2p)
*T*	0.44	0.35
%*A*	0.27	0.30
%*N*	0.19	0.25

**Table 2:** Phenotypic and genetic correlations between F1 triploid reproductive traits (total ovule number per silique (*T*); % of aborted, normal and unfertilized F2 seed (%*A*, %*N*, %*U*)); genetic correlations across F1 cross direction are also shown. Correlations between the same trait are equal to 1.00 (shown in italics)

	*T*	%*A*	%*N*	%*U*
*Phenotypic correlations*				
Maternal excess (2m:1p) traits				
*T*	*1.00*	− 0.43	0.01	0.39
%*A*		*1.00*	− 0.18	− 0.77
%*N*			*1.00*	− 0.47
%*U*				*1.00*
Paternal excess (1m:2p) traits				
*T*	*1.00*	− 0.41	− 0.04	0.37
%*A*		*1.00*	− 0.14	− 0.76
%*N*			*1.00*	− 0.54
%*U*				*1.00*
*Genetic correlations*				
Maternal excess (2m:1p) traits				
*T*	*1.00*	− 0.60	− 0.04	0.50
%*A*		*1.00*	0.05	− 0.83
%*N*			*1.00*	− 0.60
%*U*				*1.00*
Paternal excess (1m:2p) traits				
*T*	*1.00*	− 0.61	0.06	0.52
%*A*		*1.00*	− 0.21	− 0.78
%*N*			*1.00*	− 0.45
%*U*				*1.00*

Across directions	*T* (1m:2p)	%*A* (1m:2p)	%*N* (1m:2p)	%*U* (1m:2p)
T (2m:1p)	0.21	− 0.13	0.01	0.12
%*A* (2m:1p)	− 0.37	0.20	− 0.14	− 0.09
%*N* (2m:1p)	− 0.22	0.08	0.04	− 0.10
%*U* (2m:1p)	0.43	− 0.22	0.10	0.14

**Fig. 4:**
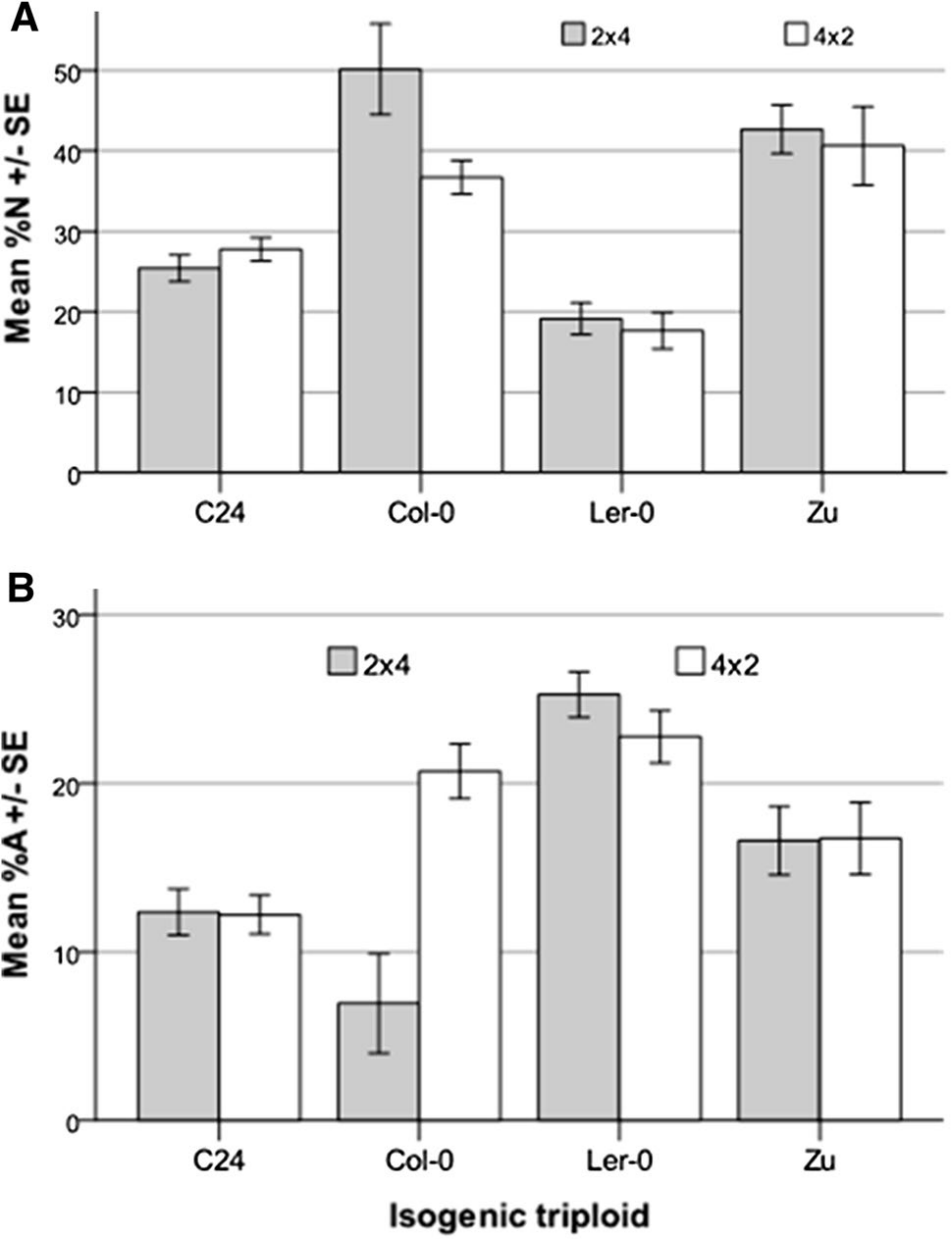
F2 abortion in seed of isogenic triploids. Mean proportions of normal (%*N*) and aborted (%*A*) F2 seeds produced when F1 isogenic triploids are allowed to self; in each case, the values from the 1m:2p F1 triploid are shown in grey, those from the 2m:1p in white; standard errors (SE) are shown

As controls, we also generated reciprocal F1 isogenic triploids in the genetic backgrounds of four accessions and measured their %*N* and %*A* (Table S1E, Fig. 4). The 1m:2p paternal excess F1 triploids in the Col-0 accession background, which experiences severe F1 seed lethality following inter-ploidy crosses (Dilkes et al. 2008), produced a high proportion of aborted F2 seeds after self-fertilization (20.7%), while the extent of F2 seed abortion in the reciprocal direction was significantly lower (6.9%, *p* < 0.001). Within the other three genetic backgrounds (C24, L*er*-0 and Zu), no statistically significant difference in F2 seed abortion was found between the reciprocal pairs of F1 triploids, indicating that grandparental-type parent-of-origin effects may not typically occur in the absence of hybridity.

To confirm that the observed post-fertilization F2 seed lethality in F1 hybrid triploid siliques is specific to effects seen in the polyploid state, %*N* and %*A* F2 seed was also determined for diploid F1 hybrids derived from reciprocal crosses between diploid L*er*-0 and five diploid accessions which form F1 triploids with particularly high or low seed viability (Wei-0, Ga-0, Pro-0, Ren-1, Fei-0). Although there was a slightly significant parent-of-origin effect on %*A* in Pro-0 (*p* < 0.05), no effect was seen in the other four accessions, and post-fertilization F2 seed abortion never exceeded 2% (Table S1D; Figure S2). We conclude that the genotypic and grandparent-of-origin effects on seed lethality in F2 seeds observed from selfed F1 hybrid triploids are principally due to parental genome dosage effects rather than being a consequence of hybrid dysgenesis.

### Natural variation in F2 seed production in paternal and maternal excess F1 triploids

As F2 seed production from selfed Arabidopsis F1 hybrid triploids is significantly influenced by the grandparental genotypes, we sought to identify the underlying genetic basis by performing a genome-wide association study (GWAS). This made use of the fact that the accessions crossed to the L*er*-0 tetraploid tester line belonged to a mapping population for which 1.2 million SNPs had been identified (Atwell et al. 2010; Horton et al. 2012). We used an Efficient Mixed-Model Association (EMMA) approach to correct for population structure (Atwell et al. 2010; Korte et al. 2012; Zhao et al. 2007) and searched for statistical associations between %*N* (*N*/*T*) in both cross directions using a genome-wide sampling of SNPs. Although many peaks were observed across all five chromosomes, applying a cut-off of *p* < 0.01 indicated that many of these were below the threshold of significance (Figure S5). However, two highly significant SNPs were found to be associated with %*N* of F2 seeds at the more stringent threshold of *p* < 10^−7^. As each of these SNPs was only associated with %*N* F2 seed production in one class of F1 hybrid triploids, we termed these loci *maternal overdose triploid* (*MOT*) and *paternal overdose triploid* (*POT*), respectively. *MOT* lies within an intron of a gene of unknown function (AT1G53640) expressed in the seed and 19 other plant structures, while *POT* is located in an intergenic region (Table S2). Neither SNP was found to be clustered with other significant or nearly significant SNPs. Although it is possible that genes in the vicinity of these SNPs are associated with aspects of F2 seed production from selfed F1 triploids, it is likely that analysis of natural variation in greater numbers of accessions will be required to more conclusively identify the causal loci. As %*N* depends upon the capacity of the F1 plant to fertilize its ovules successfully (indicated by the %*U* trait) and the subsequent chance of normal seed development [indicated by %*N*/(*N* + *A*)], we also investigated the results of GWAS on these traits, but no significant peaks were found (Figure S6). Hence, the genetic associations can be considered specific to the overall production of normal (%*N*) seeds. The excess of small *p* values in all cases could suggest that the control of triploid reproduction may be polygenic, although some residual confounding effects might also be occurring (as suggested by the *p* value distributions; Figure S7).

### Differences between selfed F1 hybrid triploids in DNA content of F2 embryos, including grandparental effects

**Fig. 5:**
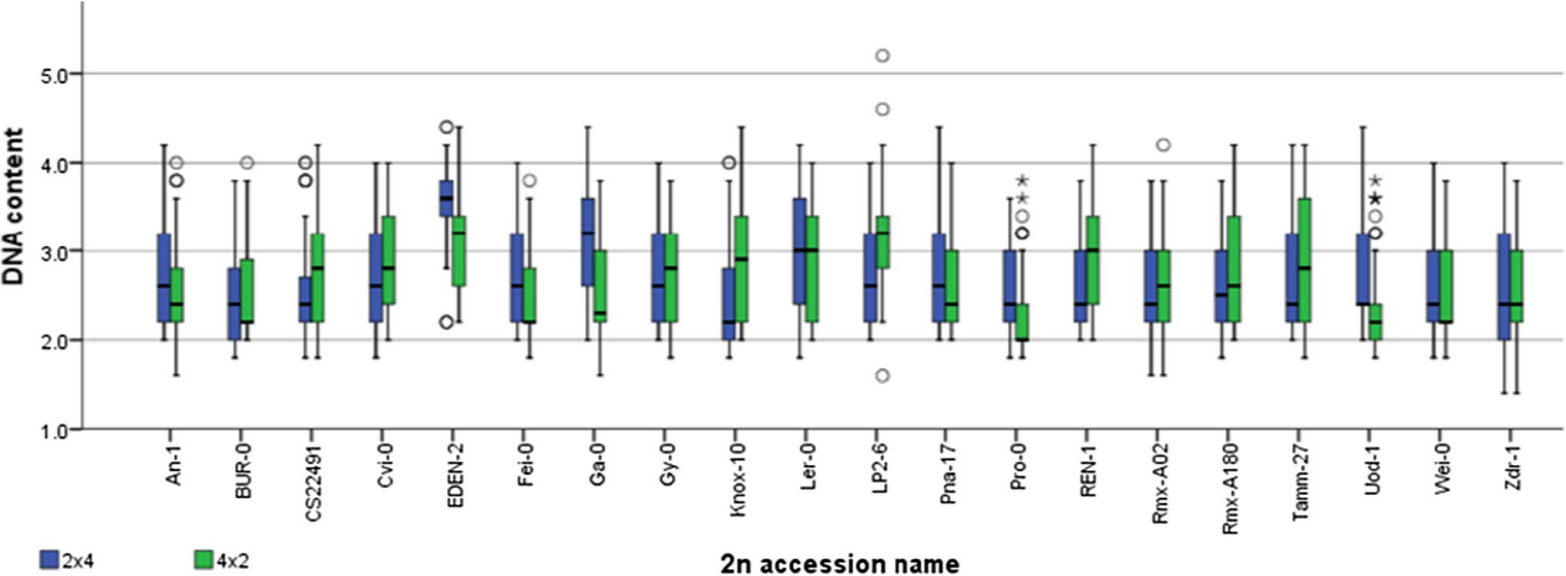
Variation in F2 embryo ploidy levels in seed of F1 hybrid triploid plants. Box plots illustrating ploidy ranges of embryos from F2 seed produced from maternal excess (2m:1p; blue) and paternal excess (1m:2p; green) F1 hybrid triploids, listed by the name of the diploid accession crossed to the L*er*-0 tetraploid line; histograms illustrating ploidy distributions are shown in Suppl. Figure 4. Outliers are indicated with voided circles

**Fig. 6:**
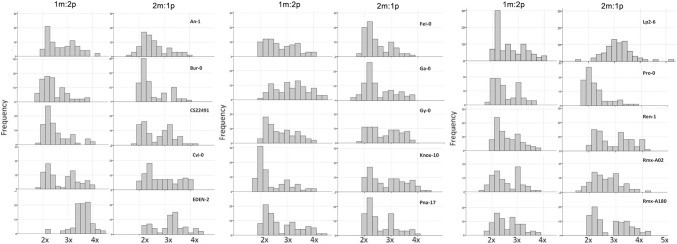
Variation in ploidy of mature F2 seed produced by selfed F1 hybrid triploids. Histograms show ploidy distributions of embryos of non-aborted mature F2 seed (*n* = 90). Distributions are listed by the name of the diploid accession crossed to the L*er*-0 tetraploid line. In each case, the distribution of F2 embryos from the paternal excess (1m:2p) triploid is shown on the left, and that of the maternal excess (2m:1p) triploid is shown on the right

Selfed F1 triploid plants produce F2 offspring that consist of a swarm of different aneuploid F2 genotypes (Henry et al. 2005). The abortion of fertilized F2 seeds from selfed F1 triploid plants could result from genome dosage imbalance effects arising from aneuploidy in the embryo, endosperm or both within the F2 seed. To investigate this, we used single-seed cytometry to compare the DNA content at maturity of populations of individual F2 seeds obtained from different selfed F1 hybrid triploids and from L*er*-0 F1 isogenic triploids (Fig. 5). The transient Arabidopsis endosperm is consumed by the embryo to leave a single-cell layer by the time of maturity (Brown et al. 1999)—hence, our data reflect the DNA content of the F2 embryos alone. The range of F2 embryo ploidy levels was highly variable across individual F2 seed progeny from different F1 hybrid triploids and included likely many aneuploids (Fig. 6), with the most variable F2 seed embryo ploidy content observed in the Lp2-6 maternal excess triploid which ranged from < 2 × (diploid) to > 5 × (pentaploid). While the DNA content distributions in some cases resembled the normal distribution predicted for the results of triploid self-pollination (Henry et al. 2009), the majority of the F2 seed embryo distributions from our panel of F1 hybrid triploids deviated considerably from this. Many were bimodal, with one peak at ~ 2–2.4 × and another at ~ 3.0–3.4 ×, consistent with previous observations of Col-0 and Col-0 X Wa-1 triploids (Henry et al. 2005). However, other F1 hybrid triploids preferentially produced offspring only with a reduced ploidy (e.g. Uod-1 in either cross direction). F1 hybrid triploids producing many F2 seed progeny with an elevated genome content (3.6–4.0 ×) were rare, although this was observed for F2 seeds produced from the paternal excess F1 triploid derived from an Eden-2 grandmother (Fig. 6), suggesting that some F1 hybrid triploid lines may trigger non-reduction effects leading to higher ploidy levels in subsequent generations. In addition, as many of these F2 seed embryo DNA content distributions vary between the pairs of genetically identical F1 hybrid triploids with maternal and paternal genome excess, we conclude that grandparent-of-origin effects also affect the degree of aneuploidy in the F2 offspring, but that this does not necessarily correlate with F2 seed abortion.

## Discussion

Polyploidy plays an important role in the evolution, speciation and reproductive success of both plants and animals (Van de Peer et al. 2017). Triploid plants occur in many populations in which diploid and tetraploid relatives can interbreed, while triploid plants can also arise via polyspermy events (Nakel et al. 2017). In some cases, this interbreeding may only be possible in one direction due to F1 seed abortion arising from genomic imbalance in the seed endosperm, typically in the paternal excess F1 triploid seed, a phenomenon termed the triploid block (Köhler et al. 2010). F1 triploids can also suffer from reproductive difficulties in subsequent generations. This arises due to difficulties in chromosome pairing: meiosis is complicated by the presence of an additional set of chromosomes which must be resolved into two poles and leads to the aneuploidy in gametes and F2 zygotes (Ramsey and Schemske 1998).

Because plants can often reproduce asexually through vegetative propagation or apomixis (Spillane et al. 2001), some triploids can be persistent and vigorous, as in the case of the highly invasive noxious weed, Japanese knotweed (te Beest et al. 2011). In addition, triploids can persist when deliberately propagated asexually by humans, as seen for vegetatively propagated crops such as banana and potato (Perrier et al. 2011). While triploid plants occur and persist in many natural populations, little is known about the effects of triploidy on sexual reproduction via seed {Husband, 2004 #34; Grossniklaus, 2001 #9}.

In this study, we have investigated the impacts of parental genome dosage effects and natural variation on F2 seed production from selfed F1 triploids using the model plant, *A. thaliana.* Triploid plants of Arabidopsis display altered phenotypes likely associated with altered expression of dosage-sensitive genes but are generally viable (Dilkes and Comai 2004; Donoghue et al. 2013; Fort et al. 2017). Parental cross direction is also important in the study of triploidy in Arabidopsis due to the occurrence of triploid block in some accessions {Köhler, 2010 #17; Borges, 2018 #72; Spillane, 2002 #39} and parent-of-origin effects on ovule number and fertility where F1 can be produced (Duszynska et al. 2013). Importantly, using reciprocal crosses between diploid and tetraploid parents of *A. thaliana*, it is possible to generate F1 triploid plants that are genetically identical, apart from inheriting two sets of chromosomes from either the maternal (maternal excess F1 triploid, 2m:1p) or paternal parent (paternal excess F1 triploid, 1m:2p). Parent-of-origin effects occur when the phenotype observed in the F1 offspring is dependent on whether a gene, chromosome set or trait is inherited from the mother or the father {Spillane, 2002 #39}. In our case, we considered whether there was any evidence for grandparental-type parent-of-origin effects where an additional chromosome set transmitted to an F1 parent by the grandmother or grandfather of an F2 seed (derived from an F1 hybrid triploid) had an effect on the F2 seed (and its embryo DNA content). In our study, each family of F2 seeds is derived from a reciprocal cross between two grandparents (L*er*-0 4 × and a 2 × accession), where the L*er*-0 4 × line remained constant as a tester line (Fig. 1). Each of these grandparental crosses resulted in an F1 hybrid triploid that was subsequently selfed to generate F2 seed, with multiple replicates used for all accessions, cross directions, plants and siliques to ensure the robustness of all data points.

### Natural variation affects F2 seed reproductive success from F1 triploid plants

To determine the impacts of genotype and cross direction on F2 seed production from F1 triploid hybrid plants, we compared the production of aborted and non-aborted (*A*) and apparently normal, non-aborted (*N*) F2 seeds from the selfed F1 hybrid triploids (the relative proportions of these among the total ovules produced by the F1 plants are termed %*A* and %*N*, respectively). We find that the %*N* of F2 seeds is greatly influenced by the genotype of the F1 hybrid triploid (Fig. 2a, b; Table 1); similarly, the genotype of the F1 hybrid triploid also influences the extent of F2 seed abortion, %*A* (Fig. 3a, b; Table 1). The isogenic test-cross controls conducted in Fig. 4 (across *n* = 15 siliques/~ 900 ovules) demonstrate that while grandparental-type parental effects on F2 seed %*N* and %*A* can occur for some genetic backgrounds (e.g. for Col-0), such parental effects are absent in most of the isogenic F1 triploid backgrounds. However, in the case of F1 hybrid triploids there are significant effects due to the presence of genetic variation between parents (*p* < 0.001 for the interaction term, Table 2). For example, the %*N* found in F2 offspring of 2m:1p isogenic Col-0 F1 triploids is 36.7%, while %*N* for 2m:1p produced by crossing 4 × L*er*-0 with 2 × Col-0 is only 23.2%, a difference which would also be significant if considered in isolation.

F2 seed abortion from selfed F1 triploids is likely a consequence of aneuploidy, which can cause imbalance of dosage-sensitive genes and so lead to post-zygotic lethality (Birchler 1993; Ramsey and Schemske 1998). Such dosage-sensitive genes may include those regulated by genomic imprinting, some of which may contribute to speciation mechanisms (Wolff et al. 2015). However, some F2 genotypes can revert to a euploid condition, possibly in cases where they inherit chromosome assortments which do not disrupt any highly dosage-sensitive genes (Freeling 2009). To investigate the hypothesis that F2 seed success from F1 triploid reproduction could be associated with possible avoidance of aneuploidy, we determined whether the embryos of %*N* F2 seed families represented euploid F2 offspring. The resulting flow cytometry data (Fig. 6) suggest that this is not necessarily the case: although all F1 hybrid triploid lines show elevated %*A*, the embryos from non-aborted F2 seed analysed by flow cytometry also had highly variable ploidy, including frequent aneuploidy. We can conclude that not all aneuploid F2 seeds abort within the silique, irrespective of whatever fitness impacts they might suffer later in the life cycle.

We also considered the possibility that natural variation in the tendency to experience triploid block effects (Köhler et al. 2010; Borges et al. 2018) in the original cross between both grandparents could contribute to subsequent parental effects on F2 seed production from selfed F1 triploids. In this scenario, triploid block “escapes” of very small numbers of 1m:2p F1 triploid seeds could alter their subsequent development as F1 plants and their ability to generate F2 seeds. Indeed, Col-0, which is known to experience a strong triploid block, does display a significant parental effect on %*A* (although not on %*N*). However, we consider that this is unlikely to be wholly responsible for the variation seen, as accessions with very strong triploid blocks could not be used for determining the strength of the parental effect as they failed to produce 1m:2p F1 hybrid triploid plants (Table S1). It is possible that there is natural variation for tolerance of aneuploidy during seed development, with more tolerant F1 plants producing less %*A* and more %*N* F2 seed, as a proportion of the total fertilized ovules. Given the trade-off that we noted between the proportion of ovules fertilized and the proportion which subsequently develop without aborting (1m:2p, *p* = 0.001; 2m:1p, *p* = 0.002), it is also possible that F1 plant vigour, and hence availability of resources to support post-fertilization seed development, could be a variable which deserves further investigation. Taken together, our results indicate that genetic variation significantly influences the ability of different F1 hybrid triploid genotypes to produce F2 seeds.

### Possible “grandparental” effects on F2 seeds derived from F1 triploid hybrids display

Previous studies of offspring of F1 triploid Arabidopsis have indicated significant effects of cross direction on F1 ovule fertility (Duszynska et al. 2013). In this study, our crossing design was set-up to also allow us to detect “grandparent-of-origin” effects on F2 seeds. We demonstrate that F2 seed traits can indeed vary due to such grandparental effects, with different proportions of normal (%*N*) and aborted (%*A*) F2 seeds arising from selfing of genetically identical reciprocal F1 hybrid triploids (Figs. 2c, 3c). Furthermore, our analysis of F2 seed embryo DNA content reveals that F1 hybrid triploids can generate F2 seeds with dramatic differences in mean DNA content depending on the genotype of the F1 hybrid triploid (Fig. 5). In addition, our measurements of the DNA content of individual F2 seeds for families comprising 90 F2 seeds from each selfed F1 hybrid triploid reveal that the DNA content distribution displays high levels of variability between F1 hybrid triploid genotypes (Fig. 6). This could indicate the existence of transgenerational grandparental effects on the F2 seeds that are manifested two generations after the initial cross between the L*er*-0 tetraploid tester line and the diploid accession. We note that a conclusive demonstration of whether these can be termed “grandparental” effects depends upon the biological basis of the traits. As noted above, some possible sources of this variation could include natural variation for tolerating aneuploidy within the original F0 grandparental genotypes; altered development of F1 seed (including possible legacy contribution of triploid block effects); and possible effects of F1 plant vigour. Elucidating this will require greater understanding of the effects of triploidy on F1 seed development, and whether such effects are gametophytic, sporophytic or a combination of both (Spillane et al. 2002; Fort et al. 2017). Gametophytic parental effects could be due to deposition of maternally or paternally derived factors in the fertilized endosperm or zygote, to dosage effects in the endosperm or due to genomic imprinting effects (Spillane et al. 2002), while sporophytic parental effects could arise from the maternal seed coat (Spillane et al. 2002; McKeown et al. 2011; Köhler et al. 2012).

The long-lasting parental effects on %*A* and %*N* are principally observed in the F2 seed set from the F1 hybrid triploids, although they can also occur in offspring of some F1 isogenic triploids as well (Fig. 4). This suggests that the direction of crossing of grandparents when an F1 triploid is being generated can have significant implications for the F2 seed set resulting from the selfed F1 triploid, with possible asymmetric consequences for gene flow via triploid bridges generated from maternal vs paternal tetraploid parents. Such control of offspring ploidy can have important consequences for the evolutionary outcomes of F1 triploid formation: viable aneuploids can act as vectors for gene flow between triploid and tetraploid populations as triploid progeny canalize towards stable diploid or tetraploid forms, while inviable aneuploids may lead to polyploidy-mediated speciation events (Soltis et al. 2004). Our data indicate that these scenarios are under genetic control and may arise in different F1 hybrid triploid genetic backgrounds of Arabidopsis.

In at least some cases, Arabidopsis F1 hybrid triploid genotypes seem to lead to high tolerance to aneuploidy in the F2 embryos, which is confirmed in this study (Fig. 6), suggesting that gene flow from naturally occurring tetraploid accessions may occur via triploid bridges. Such bridges are significant for speciation within *Senecio* [reviewed (Vallejo-Marín and Hiscock 2016)], but it remains to be determined what effects they have had on polyploid speciation within the *Arabidopsis* genus (Schmickl and Koch 2011; Novikova et al. 2018). It has been argued that “cryptic” tetraploid species—i.e. tetraploid populations which are reproductively isolated from their diploid relatives and therefore meet the criteria to be considered species, but have yet to be recognized as such—may be widespread in plants, with at least five previously unknown examples reported in 2007 (Soltis et al. 2007). This number has recently been expanded to a further 59 potential cryptic polyploid species in angiosperms (Kolář et al. 2017), and many more examples are likely to exist in groups such as ferns (Schneider et al. 2017) and potentially lycophytes (Wang et al. 2017). The presence of “genome reduction” effects in F2 Arabidopsis plants produced from F1 hybrid triploid parents with unbalanced “grandparental ploidy” towards a euploid condition suggests that introgression from tetraploids into diploids may be under genetic control and occur quickly in terms of generations.

### Insights into the control of triploid reproduction in plants

Our study has indicated that parental- and grandparental-type effects can affect production of F2 seed by F1 triploids, through mechanisms which remain to be determined. To identify the genetic architecture controlling F2 seed production, and begin the process of determining the causative mechanisms, we performed a genome-wide association study (GWAS) on %*A* and %*N*, noting that the power of this initial analysis was limited due to the relatively low sample size (Platt et al. 2010). Despite this, our results clearly indicate that the genetic architecture differs between parents (Figure S5), which agrees with the highly significant interaction term between genetic variation and cross direction found in the analysis of variation for the percentage of normal F2 seeds (%*N*) (Table 1). Although no significant SNPs were found for %*A* or %*U*, two SNPs significantly associated with the production of normal F2 seeds (%*N*) from selfed reciprocal F1 hybrid triploids were identified (Figure S4). We termed these *MOT* (*maternal overdose triploid*) and *POT* (*paternal overdose triploid*); the causative genes controlling the percentage of normal F2 seeds (%*N*) await conclusive identification. Significant peaks were found for %*N* rather than %*N*/(*A* + *N*) even though %*N* depends upon both fertilization rate and successful post-fertilization development, and it can be considered more complex than %*U*. The finding of significant peaks for %*N* would make sense if the causative loci provide general tolerance to aneuploidy, which could improve the success of both F1 gametophyte development and F2 seed development. In any case, our study indicates that the loci involved in production of normal F2 seeds (%*N*) seed from selfed F1 hybrid triploid plants may vary depending upon whether the F1 triploid was generated with an additional copy of the maternally or paternally derived genome. It should be noted that the existence of such grandparent-of-origin differences may be useful for interpreting GWAS data from other triploid plants such as banana (Nyine et al. 2016). While the causative genes remain to be determined, we note that the SNP-associated locus we termed *MOT* is located on chromosome 1, in relative vicinity to the as-yet unmapped *SDI* locus identified previously (Henry et al. 2009).

### Conclusions and future directions

We have previously argued that the effects of parental cross direction on reproductive characteristics of selfed F1 triploids could be due to epigenetic differences between maternally and paternally derived chromosomes, the action of maternally inherited cytoplasmic organelles or unusually long-lasting maternal effects, or some combination of these (Duszynska et al. 2013). Here, we have extended such investigations to demonstrate that parental effects deriving from the generation of F1 triploids can extend to the F2 seed generated from the selfed F1 triploids. We have characterized differences in F2 seed produced by selfing of genetically identical pairs of F1 hybrid triploids and detected what may represent “grandparental effects” transmitted through the growth of reciprocal F1 hybrid plants to their reproductive stage, and which manifest as differences in the F2 seed abortion, development and ploidy. We consider that such genotypic and grandparental effects could have significant implications for F1 triploid hybrids acting as “engines” of evolutionary novelty, particularly for generation of hybrid offspring that can differentially canalize over subsequent generations towards novel euploid lineages (Henry et al. 2010; Cheng et al. 2018; Thomas et al. 2006). We consider that further characterization of the genetic basis of parental effects in Arabidopsis triploid reproduction can provide important insights into the regulation of reproduction and gene flow in polyploid plants, and contribute to improved breeding programmes for polyploid crops.

## Materials and methods

### Plant material

Triploid Arabidopsis F1 inter-accession hybrids were generated as previously described (Donoghue et al. 2013; Duszynska et al. 2013) using 88 wild inbred accessions from a 96-accession mapping panel (Nordborg et al. 2005; Zhao et al. 2007) reciprocally crossed with tetraploid L*er*-0 (the gift of Prof. Ueli Grossniklaus, Cold Spring Harbor Laboratories). F1 hybrid triploid seeds were germinated, grown under identical growth conditions, tested to confirm eutriploidy and allowed to self-pollinate to generate F2 progeny.

### Reproductive phenotypes

Mature siliques were dissected with fine tweezers under a Leica MZ6 microscope, and the F2 seeds from 15 siliques (five from each of three individual plants) were scored for numbers of normally developing or aborted seeds (N, A) and for the total number of ovules whether fertilized or not (*T*). These were expressed as both proportions of the total ovule number (%*N* = *N*/*T*; %*A* = *A*/*T*) and proportions of the number of ovules which had been fertilized (*N*/(*N* + *A*), *A*/(*N* + *A*)) to distinguish between total plant fecundity and the post-fertilization fate of the F2 seeds specifically.

### Statistical analyses of heritability

Reproductive characters obtained from crossing experiments were analysed using linear mixed models in PROC Mixed in SAS (Littel et al. 1996) with accession and cross direction as fixed factors and including their interaction. In this framework, the presence of trait genetic variation is indicated by a significant accession term, parent-of-origin effects indicated by a significant direction term, and genetic variation in parent-of-origin effects by significant accession x cross direction interaction. Broad-sense heritability was estimated as the among accession variance component divided by the total phenotype variance (Falconer and Mackay 1996; Lynch and Walsh 1998) under models considering accession a random effect and splitting by cross direction. Genetic correlations among reproductive characters (in both cross directions) were estimated by the standard Pearson product–moment correlation of accession means. The significance of each genetic correlation was determined using a t test after a Z transformation of the correlation coefficient. Levels of significance for phenotypic and genetic correlations were not adjusted for multiple tests.

### Genome-wide association mapping (GWAS)

A GWAS mapping approach was used to find correlation between the measured variation in the traits (%*N*, %*U*, %*N*/(*N* + *A*)) and 1.4 million genome-wide SNP polymorphisms across the genomes of the diploid parental accessions. A mixed-model approach implemented in the programme EMMA was used to control for population structure (Atwell et al. 2010; Yu et al. 2006) using five transformations (exp, log, sqrt, sqr and none) of the phenotype values; in each case, the transformation which resulted in most Gaussian distribution was considered further. Regions of significance were defined as the distance between the nearest adjacent non-significant SNPs flanking each significant SNP (*p* < 0.01) following application of a Bonferroni threshold. Candidate genes within 120 kb of significant SNPs were identified using TAIR (http://www.arabidopsis.org/).

### Single-seed ploidy analysis

DNA content of 90 single F2 seeds per selfed F1 triploid parent was measured in relation to diploid Col-0 control seeds, as described in (Matzk et al. 2001), with modifications specific to Arabidopsis. Briefly, seeds were homogenized in a 96-deep well plate containing 80 µl of DNA extraction buffer (0.1 M citric acid monohydrate, 0.5% Tween 20, β-mercaptomethanol, pH 2.5) and three metal balls of 3 mm diameter, for 1 min. 100 µl of DNA staining buffer (Na_2_HPO_4_, 0.4 M DAPI, pH 8.5) was added, and the suspensions were filtered through a nylon filter of 30 µm mesh width. 80 µl of the filtrate and an additional 80 µl of staining buffer were mixed in a new 96-well plate. Fluorescence intensity of DAPI-stained nuclei from individual embryos was measured using a high-throughput Ploidy Analyser PAII PARTEC equipped with a multiplex 96-well plate Robby Well autoloader. Flomax software was used to analyse obtained genome content profiles (PARTEC GmbH).

#### Author contribution statement

CS conceived, designed and managed the research; DD, SS and RCB conducted the experiments; all authors analysed the data; and CS and PMcK wrote the manuscript. All authors read and approved the manuscript.

## Electronic supplementary material

Below is the link to the electronic supplementary material.

Supplemental Fig. 1 Triploid Arabidopsis hybrids have many defects in seed development. Siliques produced by selfed hybrid triploids made by crossing diploid accessions to a L*er*-0 tetraploid show high variability in the seed set. The offspring can be divided into three classes: seeds which are fertilized but abort at a subsequent stage are brown and withered (A) with different sizes depending upon when they aborted; seeds which are fertilized and appear to be viable show normal development (N); ovules which remain unfertilized (U) are small, white and withered. (PDF 84 kb)

Supplemental Fig. 2 Post-fertilization F2 seed fate following selfing of Arabidopsis F1 hybrid triploids. (A) The proportions of aborted (black bars) and normally developed (blue bars) produced by 1m:2p paternal excess F1 hybrid triploids as percentages of the total number of fertilized ovules; (B) the proportions of aborted (black bars) and normally developed (red bars) produced by 2m:1p maternal excess F1 hybrid triploids as percentages of the total number of fertilized ovules (PDF 27 kb)

Supplemental Fig. 3 Minimal parent-of-origin-dependent variation in F2 reproductive characters of F1 hybrid diploids (PDF 14 kb)

Supplemental Fig. 4 Seed viability of offspring of F1 hybrid Arabidopsis triploids is determined by parental genotype and cross direction. Diagonal matrix presenting the phenotypic and genetic correlations between %A, %N, and the total number of ovules per silique (T), split between maternal excess hybrids (2m:1p) and paternal excess hybrids (1m:2p) of F1 hybrid triploids (JPEG 67 kb)

Supplemental Fig. 5 Manhattan plots associated with natural variation in the production of F2 seed by F1 hybrid triploids. Genome-wide SNPs shown across Arabidopsis chromosomes 1–5 showing degree of association with %N in (A) the 2m:1p maternal genome excess triploids and (B) the 1m:2p paternal genome excess triploids; dotted horizontal lines represent the p value threshold; locations of the SNPs significantly associated with causative loci *MOT* and *POT* are circled (PDF 175 kb)

Supplemental Fig. 6 Manhattan plots associated with natural variation in the production of F2 seed by F1 hybrid triploids. Genome-wide SNPs shown across Arabidopsis chromosomes 1–5 showing degree of association with %U in (A) the 2m:1p maternal genome excess F1 triploids and (B) the 1m:2p paternal genome excess F1 triploids; and with %N/(N + A) in (C) the 2m:1p maternal genome excess F1 triploids and (D) the 1m:2p paternal genome excess F1 triploids (PDF 1121 kb)

Supplemental Fig. 7 log-qq plots for GWAS for production of F2 seed by F1 hybrid triploids (PDF 243 kb)

Supplemental Table 1 Variation in seed development defects in Arabidopsis triploids. (A) and (B): Total ovule number per silique (T), proportion of fertilized ovules (N + A), and proportions of Aborted and Normal seeds, expressed as percentages of the total ovule number and of the number of ovules which were fertilized (A/T and A/(A + N), N/T and N/(A + N), respectively) generated by selfed Arabidopsis triploids with either (A) a paternal genome excess (1m:2p) or (B) maternal genome excess (2m:1p), as displayed in Figs. 2 and 3, respectively; (C) differences in %N/(A + N) between seeds produced by pairs of genetically identical F1 hybrid triploids as shown in Figure S2; and %A and %N from (D) isogenic triploid and (E) hybrid diploid plants used as controls, as displayed in Fig. 5 and Figure S3, respectively. F1 triploids which could not be generated in one direction are noted as Not Determined (N.D.). (XLSX 33 kb)

Supplemental Table 2 Genes in the vicinity of *MOT* and *POT* as identified by GWAS for %N in maternal and paternal excess F1 triploids, respectively (XLS 57 kb)
