# Supplementary figures and images for: Transgenerational effects of inter-ploidy cross direction on reproduction and F2 seed development of Arabidopsis thaliana F1 hybrid triploids
Source: Plant Reprod. 2019 Mar 21;32(3):275–89. doi: 10.1007/s00497-019-00369-6 (PMC6675909; doi:10.1007/s00497-019-00369-6)

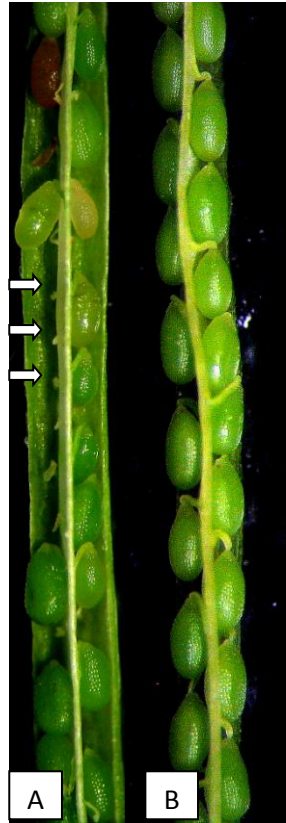

Supplement: Supplementary file 1 — Triploid Arabidopsis hybrids have many defects in seed development. Siliques produced by selfed hybrid triploids made by crossing diploid accessions to a Ler-0 tetraploid show high variability in the seed set. The offspring can be divided into three classes: seeds which are fertilized but abort at a subsequent stage are brown and withered (A) with different sizes depending upon when they aborted; seeds which are fertilized and appear to be viable show normal development (N); ovules which remain unfertilized (U) are small, white and withered. (PDF 84 kb) [file 497_2019_369_MOESM1_ESM.pdf]

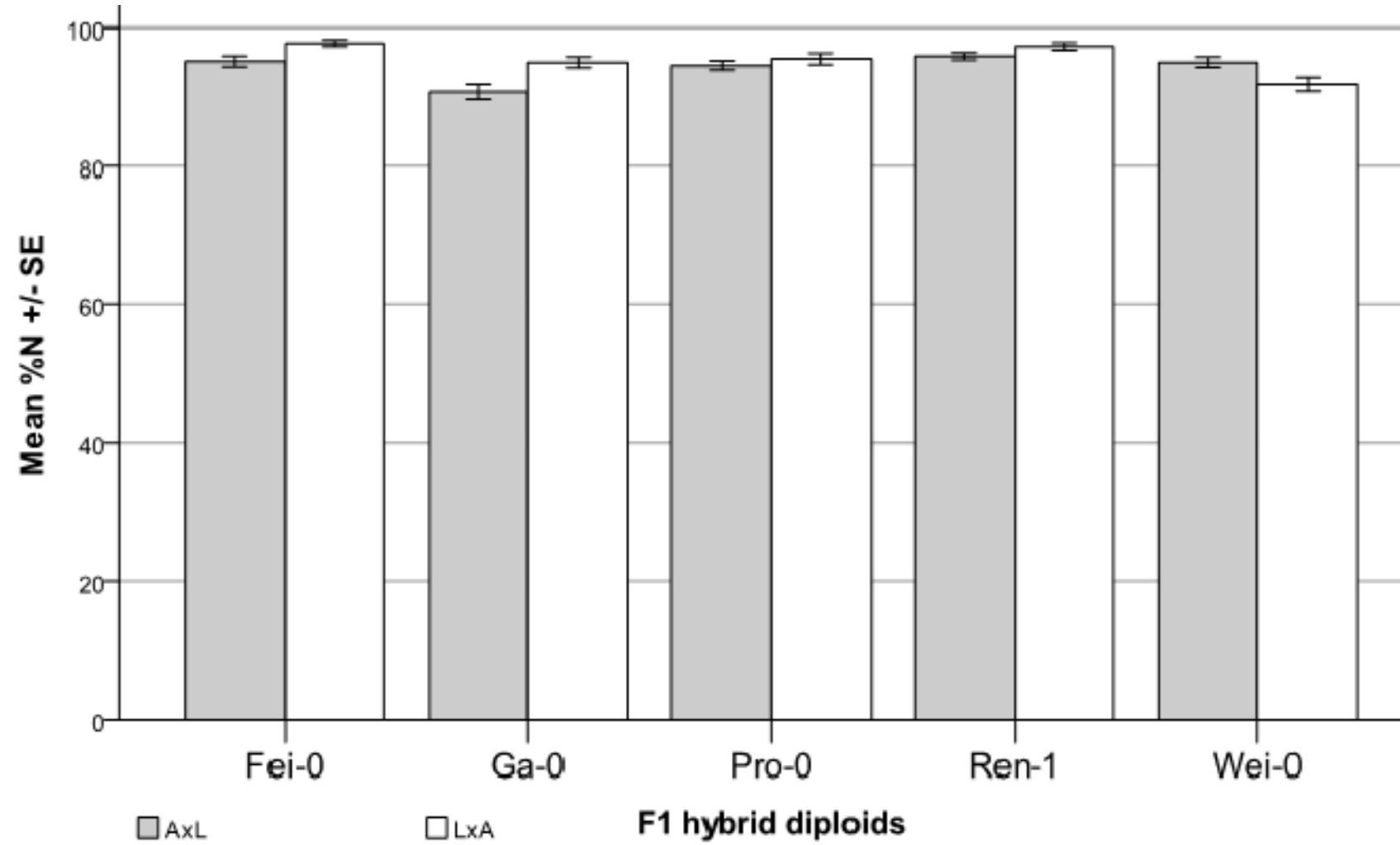

Supplement: Supplementary file 3 — Minimal parent-of-origin-dependent variation in F2 reproductive characters of F1 hybrid diploids (PDF 14 kb) [file 497_2019_369_MOESM3_ESM.pdf]

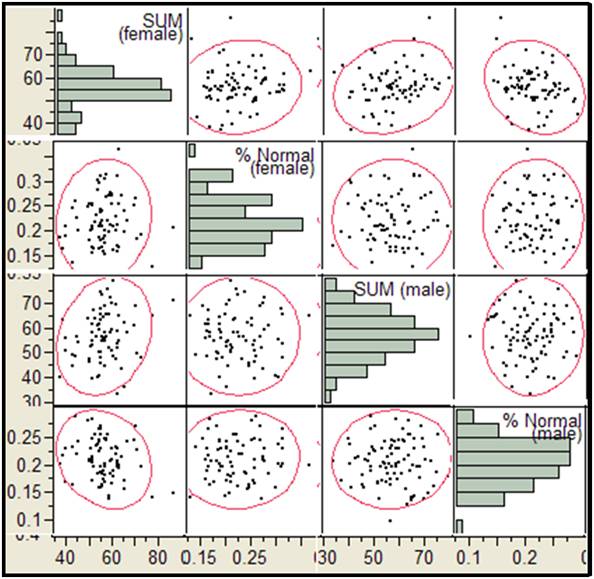

Supplement: Supplementary file 4 — Seed viability of offspring of F1 hybrid Arabidopsis triploids is determined by parental genotype and cross direction. Diagonal matrix presenting the phenotypic and genetic correlations between %A, %N, and the total number of ovules per silique (T), split between maternal excess hybrids (2m:1p) and paternal excess hybrids (1m:2p) of F1 hybrid triploids (JPEG 67 kb) [file 497_2019_369_MOESM4_ESM.jpg]

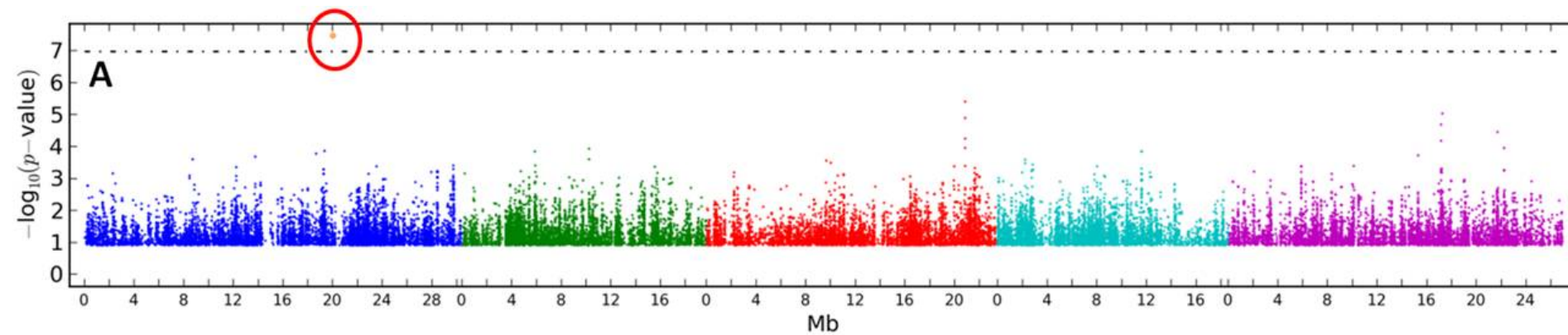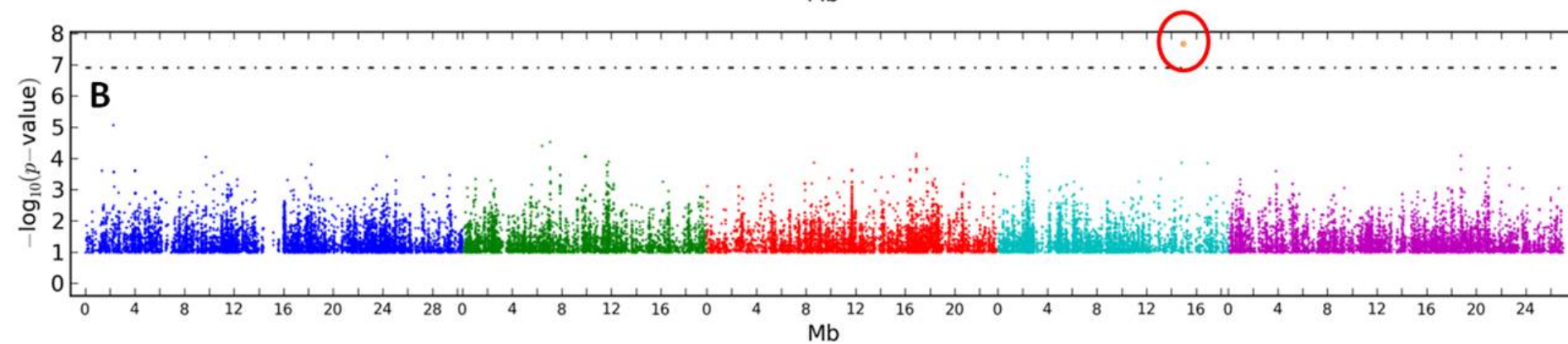

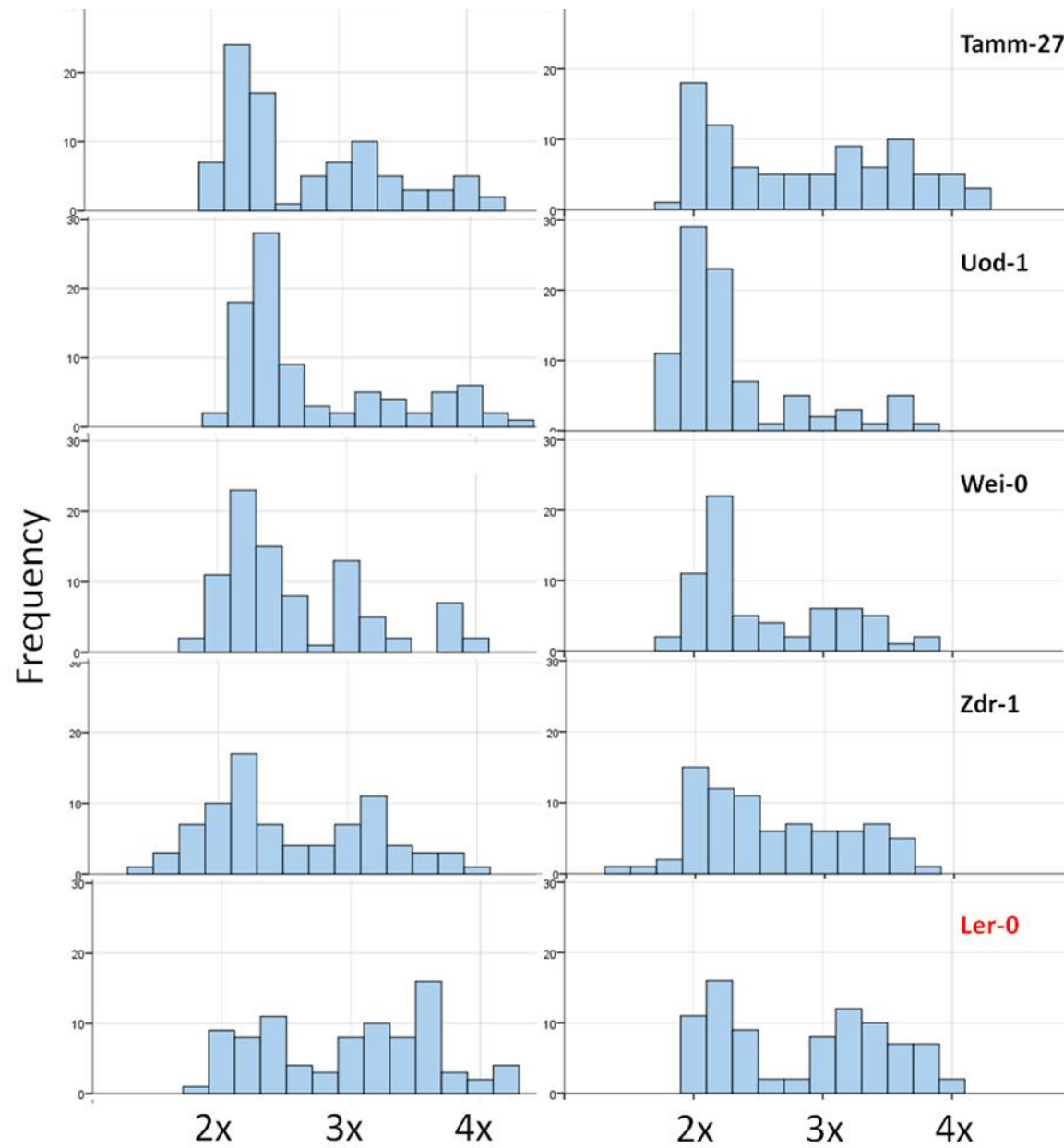

Supplement: Supplementary file 5 — Manhattan plots associated with natural variation in the production of F2 seed by F1 hybrid triploids. Genome-wide SNPs shown across Arabidopsis chromosomes 1–5 showing degree of association with %N in (A) the 2m:1p maternal genome excess triploids and (B) the 1m:2p paternal genome excess triploids; dotted horizontal lines represent the p value threshold; locations of the SNPs significantly associated with causative loci MOT and POT are circled (PDF 175 kb) [file 497_2019_369_MOESM5_ESM.pdf]

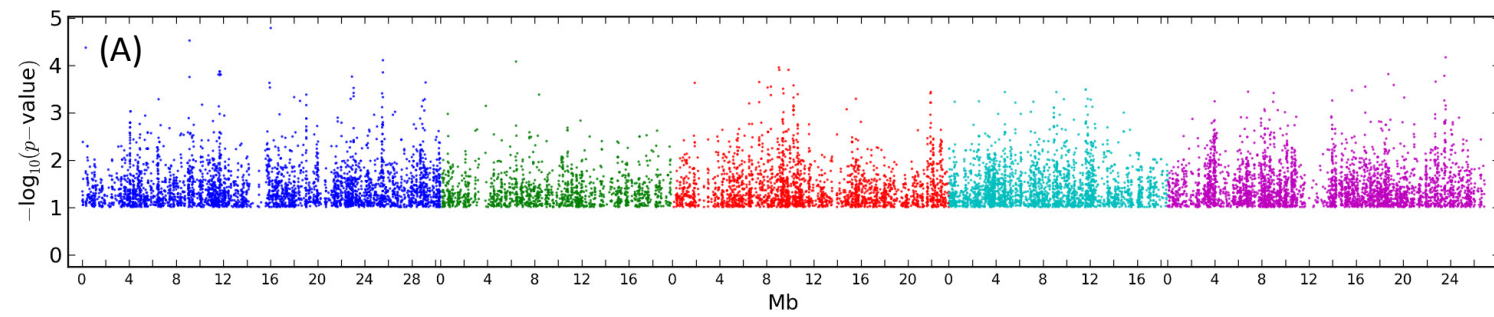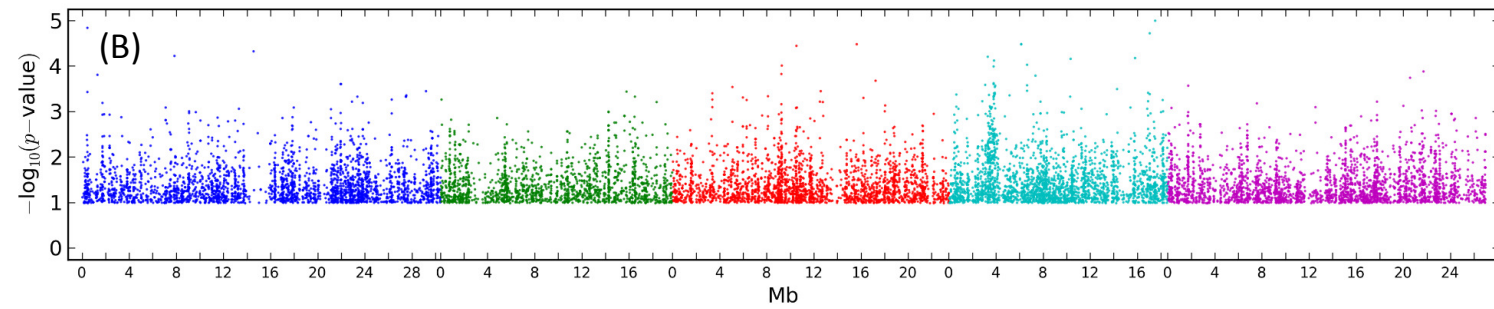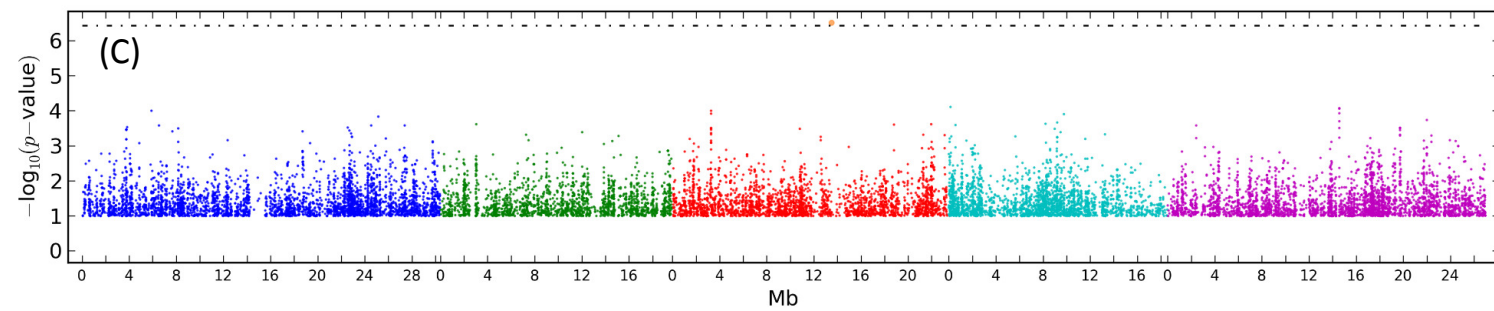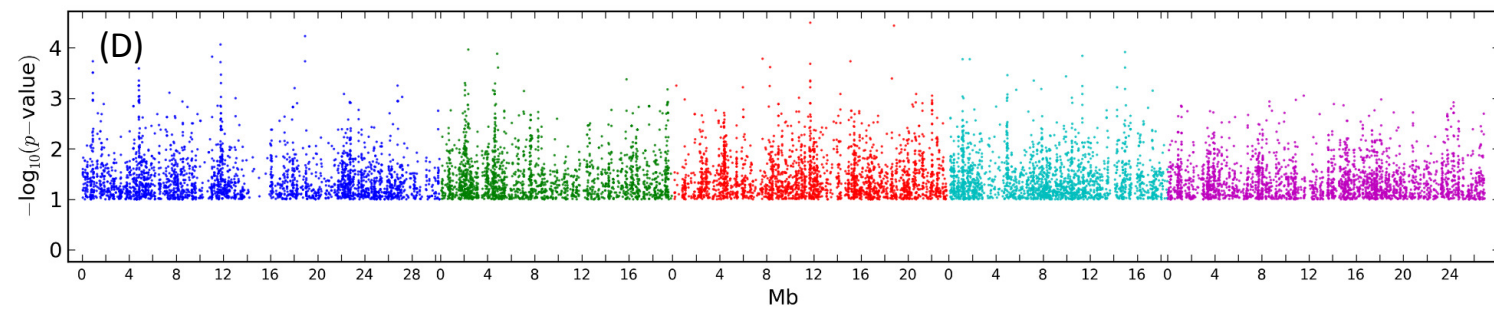

Supplement: Supplementary file 6 — Manhattan plots associated with natural variation in the production of F2 seed by F1 hybrid triploids. Genome-wide SNPs shown across Arabidopsis chromosomes 1–5 showing degree of association with %U in (A) the 2m:1p maternal genome excess F1 triploids and (B) the 1m:2p paternal genome excess F1 triploids; and with %N/(N + A) in (C) the 2m:1p maternal genome excess F1 triploids and (D) the 1m:2p paternal genome excess F1 triploids (PDF 1121 kb) [file 497_2019_369_MOESM6_ESM.pdf]

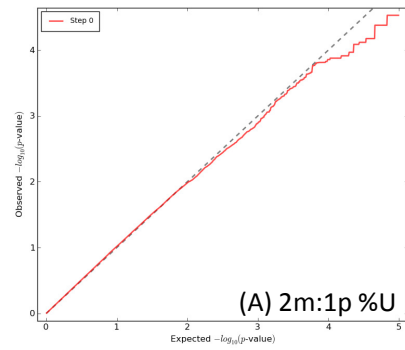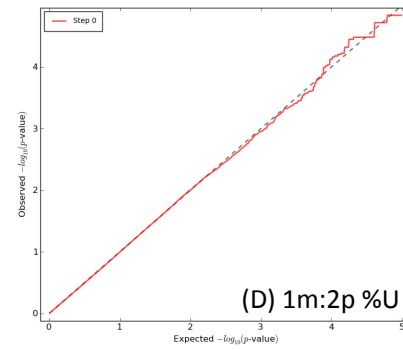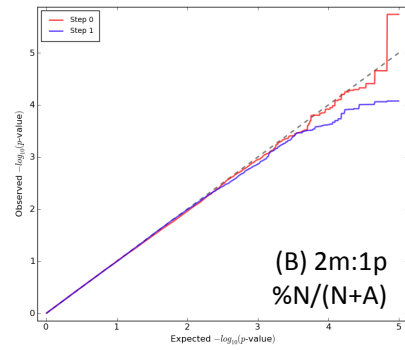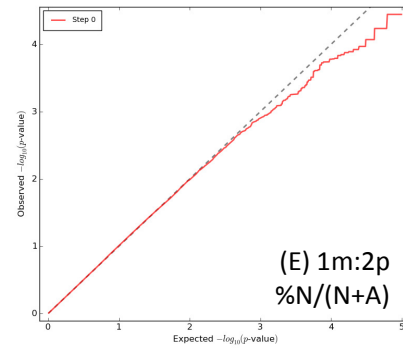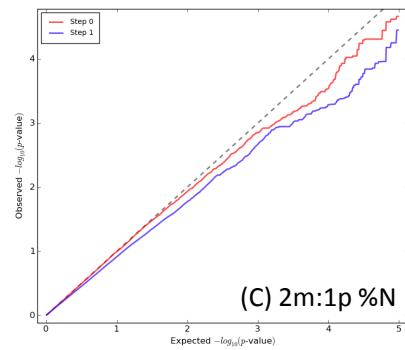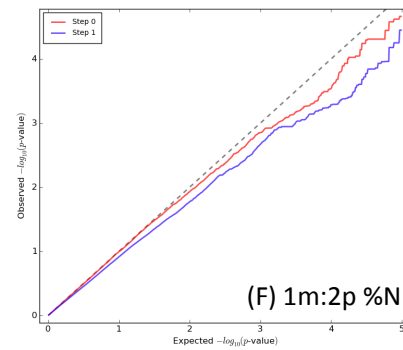

Supplement: Supplementary file 7 — log-qq plots for GWAS for production of F2 seed by F1 hybrid triploids (PDF 243 kb) [file 497_2019_369_MOESM7_ESM.pdf]
