# Supplementary material for: Transgenerational effects of inter-ploidy cross direction on reproduction and F2 seed development of Arabidopsis thaliana F1 hybrid triploids
Source: Plant Reprod. 2019 Mar 21;32(3):275–89. doi: 10.1007/s00497-019-00369-6 (PMC6675909; doi:10.1007/s00497-019-00369-6)
Supplement: Supplementary file 2 — Post-fertilization F2 seed fate following selfing of Arabidopsis F1 hybrid triploids. (A) The proportions of aborted (black bars) and normally developed (blue bars) produced by 1m:2p paternal excess F1 hybrid triploids as percentages of the total number of fertilized ovules; (B) the proportions of aborted (black bars) and normally developed (red bars) produced by 2m:1p maternal excess F1 hybrid triploids as percentages of the total number of fertilized ovules (PDF 27 kb) [file 497_2019_369_MOESM2_ESM.pdf]

(A)

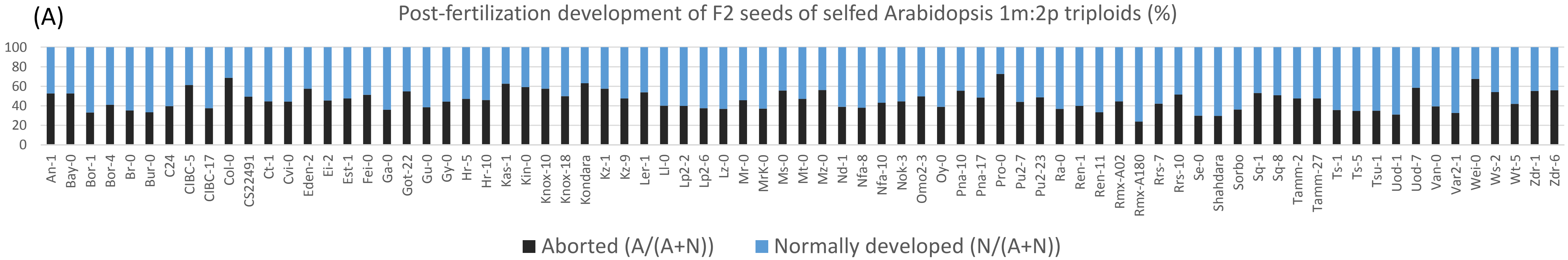
Post-fertilization development of F2 seeds of selfed Arabidopsis 1m:2p triploids (%)
100
80
60
40
20
0
An-1 Bay-0 Bor-1 Bor-4 Br-0 Bur-0 C24 CIBC-5 CIBC-17 Col-0 CS22491 Ct-1 Cvi-0 Eden-2 Ei-2 Est-1 Fei-0 Ga-0 Got-22 Gu-0 Gy-0 Hr-5 Hr-10 Kas-1 Kin-0 Knox-10 Knox-18 Kondara Kz-1 Kz-9 Ler-1 Ll-0 Lp2-2 Lp2-6 Lz-0 Mr-0 MrK-0 Ms-0 Mt-0 Mz-0 Nd-1 Nfa-8 Nfa-10 Nok-3 Omo2-3 Oy-0 Pna-10 Pna-17 Pro-0 Pu2-7 Pu2-23 Ra-0 Ren-1 Ren-11 Rmx-A02 Rmx-A180 Rrs-7 Rrs-10 Se-0 Shahdara Sorbo Sq-1 Sq-8 Tamm-2 Tamm-27 Ts-1 Ts-5 Tsu-1 Uod-1 Uod-7 Van-0 Var2-1 Wei-0 Ws-2 Wt-5 Zdr-1 Zdr-6
Aborted (A/(A+N))
Normally developed (N/(A+N))

(B)

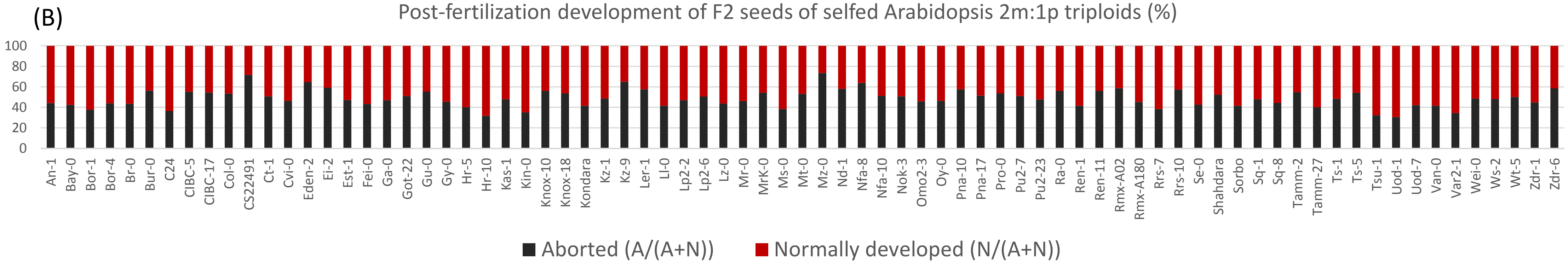
Post-fertilization development of F2 seeds of selfed Arabidopsis 2m:1p triploids (%)
100
80
60
40
20
0
An-1 Bay-0 Bor-1 Bor-4 Br-0 Bur-0 C24 CIBC-5 CIBC-17 Col-0 CS22491 Ct-1 Cvi-0 Eden-2 Ei-2 Est-1 Fei-0 Ga-0 Got-22 Gu-0 Gy-0 Hr-5 Hr-10 Kas-1 Kin-0 Knox-10 Knox-18 Kondara Kz-1 Kz-9 Ler-1 Ll-0 Lp2-2 Lp2-6 Lz-0 Mr-0 MrK-0 Ms-0 Mt-0 Mz-0 Nd-1 Nfa-8 Nfa-10 Nok-3 Omo2-3 Oy-0 Pna-10 Pna-17 Pro-0 Pu2-7 Pu2-23 Ra-0 Ren-1 Ren-11 Rmx-A02 Rmx-A180 Rrs-7 Rrs-10 Se-0 Shahdara Sorbo Sq-1 Sq-8 Tamm-2 Tamm-27 Ts-1 Ts-5 Tsu-1 Uod-1 Uod-7 Van-0 Var2-1 Wei-0 Ws-2 Wt-5 Zdr-1 Zdr-6
Aborted (A/(A+N))
Normally developed (N/(A+N))
